# Domestic Larval Control Practices and Malaria Prevalence among Under-Five Children in Burkina Faso

**DOI:** 10.1371/journal.pone.0141784

**Published:** 2015-10-30

**Authors:** Souleymane Diabaté, Thomas Druetz, Tiéba Millogo, Antarou Ly, Federica Fregonese, Seni Kouanda, Slim Haddad

**Affiliations:** 1 Faculty of Medicine, Laval University, Québec, QC, Canada; 2 CHU de Québec Research Center, Saint-Sacrement Hospital, Québec, QC, Canada; 3 University of Montreal Hospital Research Centre (CRCHUM), Montreal, Quebec, Canada; 4 Institut Africain de Santé Publique, Ouagadougou, Burkina Faso; 5 Institut de Recherche en Sciences de la Santé (IRSS) du CNRST, Ouagadougou, Burkina Faso; Johns Hopkins Bloomberg School of Public Health, UNITED STATES

## Abstract

**Introduction:**

Larval source management has contributed to malaria decline over the past years. However, little is known about the impact of larval control practices undertaken at the household level on malaria transmission.

**Methods:**

The study was conducted in Kaya health district after the 2010 mass distribution of insecticide treated-nets and the initiation of malaria awareness campaigns in Burkina Faso. The aim was to (i) estimate the level of domestic larval control practices (cleaning of the house and its surroundings, eradication of larval sources, and elimination of hollow objects that might collect water); (ii) identify key determinants; and (iii) explore the structural relationships between these practices, participation in awareness-raising activities and mothers’ knowledge/attitudes/practices, and malaria prevalence among under-five children.

**Results:**

Overall, 2004 households were surveyed and 1,705 under-five children were examined. Half of the mothers undertook at least one action to control larval proliferation. Mothers who had gone to school had better knowledge about malaria and were more likely to undertake domestic larval control practices. Living in highly exposed rural areas significantly decreased the odds of undertaking larval control actions. Mothers’ participation in malaria information sessions increased the adoption of vector control actions and bednet use. Malaria prevalence was statistically lower among children in households where mothers had undertaken at least one vector control action or used bed-nets. There was a 0.16 standard deviation decrease in malaria prevalence for every standard deviation increase in vector control practices. The effect of bednet use on malaria prevalence was of the same magnitude.

**Conclusion:**

Cleaning the house and its surroundings, eradicating breeding sites, and eliminating hollow objects that might collect water play a substantial role in preventing malaria among under-five. There is a need for national malaria control programs to include or reinforce training activities for community health workers aimed at promoting domestic larval control practices.

## Introduction

Burkina Faso’s national program for malaria control initiated, in 2010, countrywide distributions of insecticide-treated nets (ITNs) and community case management [1–2], thereby expanding previous actions of limited scale and reinforcing preventive interventions implemented over the previous decade. Prevention activities within the program include promotion of ITN use, indoor residual spraying (IRS), environmental sanitation, destruction of larval sites, and control of larval proliferation [2].

Larval source management resulted in better control of *Anopheles gambiae* in Brazil and Egypt in the mid-1900s [3–4] and eventually contributed to the observed decline in malaria morbidity and mortality in sub-Saharan Africa [5–7]. In Burkina Faso, the fight against breeding habitats relies on both collective environmental actions and the efforts of households [2, 8]. At the collective level, the national malaria control program works with local communities and health authorities to carry out environmental clean-up activities, targeting larval sites and standing bodies of water such as marshes, lakes, and large pools of water located around inhabited areas. Fundamentally, however, the control of breeding habitats depends on providing information to households and raising their awareness of the need to destroy larval sites in their immediate surroundings. Information and education on malaria transmission are essentially relayed by mass media, community radio channels, and community health workers, whose mandate includes regular sessions in the villages/urban sectors and visits to families [2]. Health workers plan a monthly schedule of visits to families living in the village or neighbourhood for which they are responsible. In these visits, they transmit information on the illness and its causes, teach mothers about malaria prevention, and treat episodes of fever. Community health workers advise mothers to keep their homes and yards clean; to cover all containers, vases, or wells containing water; to change the water meant for domestic use every 24 hours; and to eliminate any insect breeding sites both within the home and in the area immediately surrounding it, i.e., any places or items where stagnant water might collect, such as animal hoof prints, old tires, puddles of water, and flower pots [8].

Dirty domestic environments, waste containers, and small open water-containing breeding sites such as puddles, foot/hoof prints, and tire tracks contribute to mosquito proliferation [9–14]. As such, encouraging mothers to maintain a clean and safe domestic environment, and thereby to create conditions unfavourable to vector breeding, may be considered a “habitat manipulation” strategy, as recommended by WHO for larval source management [7].

Even though larval source management often includes community-based actions, more studies are needed on its outcomes at the household level [15–18]. The literature has very little to say regarding the reduction of malaria transmission attributable to household actions—which include, as in Burkina Faso, regular yard cleaning, protection of water containers, and elimination of insect breeding sites. In Ethiopia, for instance, Abate et al. found no significant difference in malaria prevalence between respondents of selected households who regularly drained stagnant water and those who did not [19]. There are also questions about how household-level larval control practices are related to household characteristics, or to individual knowledge and attitudes about malaria transmission, prevention, or treatment.

The aim of this study was to provide some preliminary answers to these questions, in a context where significantly more resources are devoted to promoting use of bednets than to promoting larval control practices at the household level. The first objective was to describe household practices with regard to breeding habitats soon (six months) after the initiation of awareness campaigns by community health workers. The study focused on three specific actions targeted by the national malaria control program that are under mothers’ responsibility: cleaning of the house and its surroundings, eradication of larval sources around the house, and elimination of hollow objects that might collect water. The second objective was to identify key household and individual factors associated with domestic larval control actions, to be able to determine the profiles of households that were more or less likely to adopt the recommended measures. The third objective was to explore the structural relationships between the adoption of these practices by mothers and: 1) participation in awareness-raising activities; 2) mothers’ knowledge, attitudes, and practices about malaria transmission and protection; and 3) malaria prevalence among under-five children. This article focuses on mothers because, in Burkina Faso, they are in charge of cleaning of the house and its surroundings, eradicating larval sources around the house, and eliminating hollow objects. This is why mothers are particularly targeted by information and awareness-raising interventions in Burkina Faso.

## Methodology

### Study site

The study was part of the team’s research program in the Kaya health district in North-Central Burkina Faso [20]. The survey was conducted in August 2011 during the period of highest malaria transmission in the rainy season, with a random sample of 2,004 households drawn from the panel of households covered by the population observatory of the Kaya Health and Demographic Surveillance System (Kaya HDSS, Kaya Health District) [20]. The households were selected from 18 urban sectors and villages located within a radius of 20 kilometres around Kaya city. Households were stratified according to geographic area (urban and rural areas). The sampling process is detailed elsewhere [21]. The interviews were conducted with mothers, or with the persons looking after the children in the mothers’ absence, in their homes.

### Data collection and variable definitions

Household and individual characteristics were extracted from the general database of the HDSS. Information related to mothers’ knowledge, attitudes, and practices was gathered through interviews, using a questionnaire derived from the Malaria Behavior Change Communication (BCC) Indicators Reference Guide [22]. To explore clean and safe domestic environment practices, respondents were asked an open-ended question about what they usually did to protect their family members from malaria. Many responses were possible, mostly having to do with cleaning the house and its surroundings, eradicating larval sites, and eliminating hollow objects (open objects containing water or able to retain water). With regard to knowledge and attitudes, mothers were asked whether they believed that: 1) malaria is caused by a mosquito bite; 2) malaria is caused by rain; 3) fever is a sign of malaria; and 4) malaria is a serious illness. One question explored their participation in any type of information session on malaria conducted by community health workers over a recall period of three months.

Information was gathered on each child in the household. Mothers were asked whether the child had slept under a bednet the night preceding the survey or had experienced an episode of illness or fever in the preceding 14 days. Rapid diagnostic tests (RDT; CareStart PAN pLDH tests; Access Bio, Inc., New Jersey, USA [23]) were performed to estimate malaria morbidity.

### Conceptual framework for the analysis of structural relationships

The structural relationships and hypotheses were based on the literature review conducted [24–28] and the authors’ understanding of causal relationships. The causal model included two exogenous and six endogenous variables. Exogenous variables were: mother’s schooling and household setting. Endogenous variables related to the child were: occurrence of an episode of illness in the previous two weeks, having slept the previous night under a bednet, and presence of malaria (confirmed by RDT). Endogenous variables related to the child’s mother were: knowledge about malaria, participation in information sessions, and adoption of domestic larval control actions. Exogenous variables in a causal model or a casual system are factors whose values do not depend on the states of other variables in the system of equations. They are assumed to share no cause with other variables in the system of equations (their values are determined by variables outside the causal system under study); otherwise they are said to be endogenous. Endogenous variables can be both cause and effect variables [29–31]. The following causal relationships were hypothesized: 1) malaria transmission is directly inhibited by domestic larval control actions and ITN use; 2) domestic larval control actions and ITN use are both influenced by mothers’ education, knowledge, and participation in information sessions; 3) better knowledge and increased participation are related to higher education levels; 4) participation in information sessions enhances knowledge; 5) attendance at information sessions and larval control actions are limited in highly exposed rural areas; 6) children undergoing an episode of illness tend to be placed under a bednet for sleeping; and 7) episodes of illness are more frequent in highly exposed rural areas than in other rural areas.

### Data analyses

The sample characteristics and larval control practices were described according to four residential locations: 1) so-called high exposure rural areas (rural settings with large or dense streams, lakes, and/or water reservoirs); 2) so-called rural “normal” areas (rural settings not close to large water reservoirs); 3) peri-urban neighbourhoods (former rural areas that have been absorbed into the city but with still limited public infrastructure and facilities); and 4) urban areas. Logistic regressions were used to explore determinants of mothers’ and households’ domestic larval control actions.

Structural relationships were studied through structural equation modelling (Generalized Structural Estimation Model routine in STATA 13 [32]), taking into consideration the multilevel structure of data (children nested within households). The model included seven structural equations corresponding to as many endogenous and exogenous variables. Standardized logistic regression coefficients were calculated and provided in addition to the unstandardized logistic regression coefficients and the odds ratios. Relationship strengths between the dependent variable and different independent variables measured in different units were compared directly using standardized logistic regression coefficients [33].

The data were analyzed anonymously. The study was approved by the Ethics Committee of the University of Montreal Hospital Research Centre and the Health Research Ethics Committee in Burkina Faso. This work was carried out with a grant from the Canadian Institutes of Health Research (CIHR, grant # GIR-229157). Data were used in conformity with the Kaya HDSS policy (authorization 1KH004-2015).

## Results

### Sample and variables

**Fig 1:**
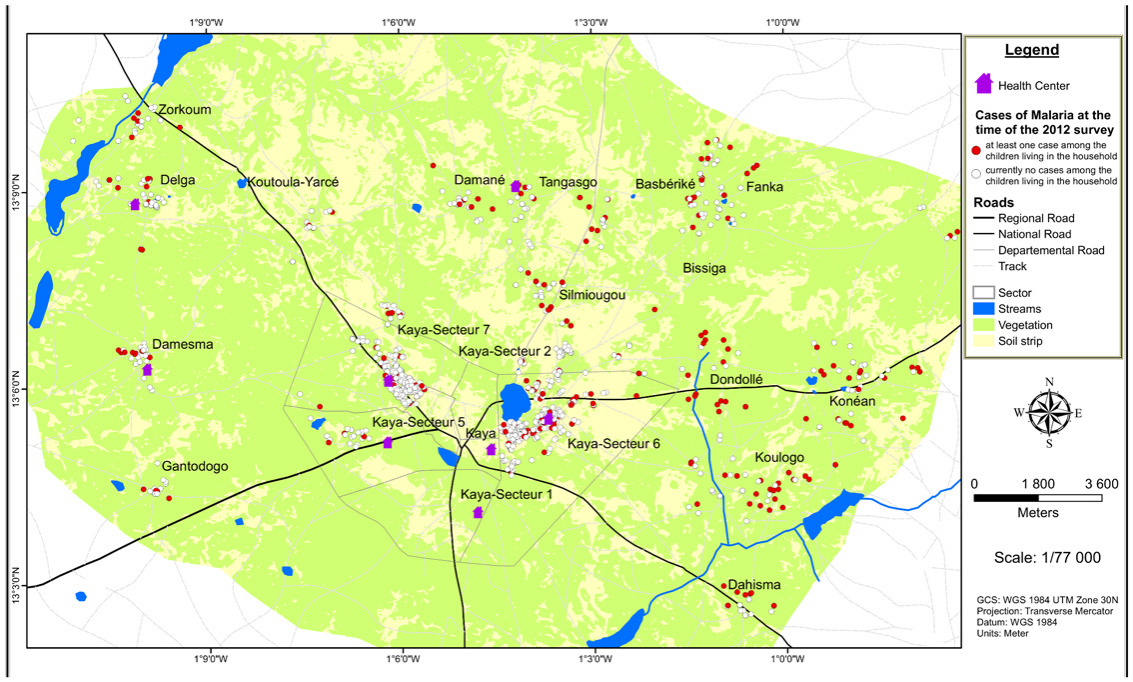
Distribution of the households surveyed. A total of 2,004 households were surveyed in 17 rural and urban sectors within a radius of 20 kilometres in the Kaya Health District, North-Central Burkina Faso. The area includes streams and stretches of stagnant water, and cases of malaria in children under five are found throughout the area.

In the 2,004 households surveyed, there were 1,906 children under five years old. Of these, 1,705 (89.5%) were present when the surveyors visited the households (Table 1 and Fig 1).

**Table 1:** Characteristics of villages and urban sectors.

Villages/urban sectors	Location	Population	Number of households	Households surveyed (%)^†^	Under-five children	Under-five children present during the interview (%)^‡^	Malaria prevalence (95% confidence interval) ^₱^
Sector 6	Peri-urban/Urban	5459	908	675 (0.74)	616	549 (0.89)	0.19 (0.16–0.23)
Sector 7	Peri-urban/Urban	3458	563	483 (0.86)	367	339 (0.92)	0.09 (0.06–0.13)
Koulgo	Rural high exposure	1327	189	75(0.40)	90	81 (0.90)	0.51 (0.40–0.63)
Dahisma	Rural high exposure	833	111	36 (0.32)	47	41 (0.87)	0.48 (0.32–0.64)
Dondolé	Rural normal exposure	887	125	49 (0.39)	45	41 (0.91)	0.58 (0.42–0.73)
Konean	Rural normal exposure	2293	297	93 (0.31)	111	90 (0.81)	0.52 (0.41–0.62)
Fanka	Rural normal exposure	2210	265	97 (0.37)	131	114 (0.87)	0.33 (0.24–0.42)
Silmigou	Rural normal exposure	1754	246	86 (0.35)	93	79 (0.85)	0.35 (0.25–0.47)
Tangasgo	Rural normal exposure	718	110	43 (0.39)	43	38 (0.88)	0.21 (0.09–0.37)
Damané	Rural normal exposure	968	132	43 (0.33)	48	45 (0.94)	0.24 (0.12–0.39)
Zablo	Rural normal exposure	336	51	24 (0.37)	25	24 (0.96)	0.29 (0.12–0.51)
Delga	Rural high exposure	1306	199	91 (0.46)	95	78 (0.82)	0.38 (0.27–0.50)
Zorkoum	Rural high exposure	1009	166	51 (0.31)	36	36 (1.00)	0.30 (0.16–0.48)
Damesma	Rural normal exposure	1066	151	55 (0.36)	59	54 (0.92)	0.31 (0.19–0.45)
Gantogdo	Rural normal exposure	487	71	29 (0.41)	21	20 (0.95)	0.40 (0.19–0.63)
Tifou	Peri-urban/Rural normal exposure	928	153	61 (0.40)	69	66 (0.96)	0.15 (0.07–0.26)
Tiwega	Rural normal exposure	760	123	13 (0.11)	10	10 (1.00)	0.30 (0.06–0.65)
**Total**		**25,799**	**3,860**	**2,004 (0.52)**	**1906**	**1,705 (0.89)**	**0.26 (0.23–0.28)**

^†^Proportion of households surveyed,

^‡^Proportion of under-five children present during the interview;

^₱^Among under-five children present in the household during the interview; Rural high exposure: rural areas with large streams, lakes, and/or stagnant water reservoirs

The proportion of mothers who had gone to school was very low, particularly in rural and peri-urban areas. Few mothers had participated in information sessions on malaria. The majority of respondents, irrespective of household location, had good knowledge about malaria. ITN use was significantly lower in urban households than in rural and peri-urban households. One child in five had a positive RDT for malaria (Table 2).

**Table 2:** Main variables and characteristics of households, mothers and under-five children.

Estimates (proportion, unless otherwise specified)	Overall	Rural high exposure^†^	Rural normal exposure	Rural normal exposure	Urban
**Households (N)**	2,004	253	564	247	940
Number of members in household: mean (SD)	6.9 (3.9)	**7.3 (3.9)**	**7.5 (4.0)**	**6.7 (3.9)**	**6.4 (3.8)**
Mothers’ age in years: mean (SD)	37.4 (13.0)	**38.9 (13.5)**	**38.9 (13.3)**	**36.5 (13.6)**	**36.4 (12.3)**
Mothers who had gone to school (at least primary school)	12.5	**3.4**	**3.3**	**7.1**	**22.6**
Households owning agricultural land	52.2	**73.1**	**78.2**	**59.5**	**29.0**
Households owning cattle^‡^	74.0	**90.9**	**93.3**	**83.0**	**55.4**
Private toilets at home	57.4	**29.7**	**28.3**	**44.1**	**95.4**
Potable water at home	46.6	**15.7**	**13.9**	**34.8**	**88.3**
**Mothers’ participation in malaria information sessions**	4.6	**6.7**	**7.6**	**3.2**	**2.6**
**Mothers’ knowledge about malaria**					
Malaria is caused by mosquito bites	97.7	99.2	97.2	96.8	97.9
Malaria is caused by rain	87.8	**85.0**	**83.7**	**83.4**	**92.2**
Malaria is a serious illness	96.6	96.8	96.6	98.0	96.2
Fever is a sign of malaria	91.7	92.5	92.4	94.7	90.3
Proportion of mothers answering all four questions correctly	77.1	77.5	73.4	76.9	79.3
**Under-five children present in the household during the interview (number)**	**1,705**	**236**	**547**	**247**	**675**
Age in years: mean (SD)	2.0 (1.3)	2.1 (1.3)	2.0 (1.3)	2.0 (1.4)	2.0 (1.4)
Female	50.2	48.3	51.7	49.0	49.9
Slept the previous night under an ITN	66.2	**65.7**	**72.0**	**65.2**	**61.9**
Episode of illness reported in the past 2 weeks	30.9	37.7	26.0	27.1	33.8
Malaria prevalence^₱^	26.0	**43.6**	**36.2**	**21.5**	**13.3**

ITN, Insecticide-treated net; In bold, p <0.05 (differences between households’ locations: chi square for proportions and Kruskal-Wallis test for medians);

^†^Rural high exposure, rural areas with large streams, lakes and/or stagnant water reservoirs;

^‡^4,811 ITNs were observed;

^₱^Positive rapid diagnostic tests.

**Fig 2:**
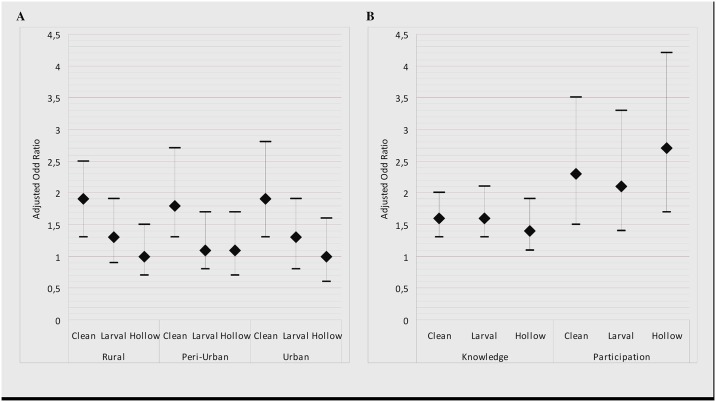
Relationship between larval control practices and household location, mothers’ knowledge and participation in malaria information sessions. A: Reference = rural highly exposed to malaria. A and B: Clean = cleaning of the house and its surroundings; Larval = eradication of larval sites; Hollow = elimination of hollow objects able to contain water. All models are adjusted for household size, ownership of cattle and/or agricultural land, access to potable water and/or to private toilets. B: Knowledge = mothers’ knowledge about malaria; Participation = participation in information session.

Cleaning the house and its surroundings was the most common larval control action reported by respondents (Table 3). The proportion of respondents who reported this practice was significantly lower for households in highly exposed rural settings compared to other areas. There was no difference between residential areas with regard to levels of eradication of larval sites and elimination of hollow objects. Logistic regression models exploring the determinants of domestic larval control actions showed that cleaning the house and its surroundings was practised less often in rural settings highly exposed to malaria than in others areas (Fig 2A). Eradication of larval sites and elimination of hollow objects were homogeneous across residential areas. The odds of undertaking any type of larval control action were significantly higher among mothers who correctly answered all four knowledge questions (adjusted odds ratio between 1.4 and 1.6, p <0.05) and among those who participated in information sessions on malaria, compared to their counterparts (adjusted odds ratio between 2.1 and 2.3, p <0.05; Fig 2B). Overall, living in households located outside highly exposed rural areas, better knowledge, and participation in information sessions were significantly associated with the practice of at least one of these three domestic larval control actions (p <0.05; not presented).

**Table 3:** Prevalence of larval control practices undertaken by mothers in the 2,004 households surveyed.

Action (proportion)	Overall	Rural high exposure^†^	Rural normal exposure	Peri-urban	Urban
**Type of action undertaken**					
Cleaning of house and surroundings	40.6	**31.2**	**45.2**	**42.9**	**39.7**
Eradication of larval sites	28.3	24.5	30.0	26.3	28.9
Elimination of hollow objects	20.3	19.4	19.9	20.2	20.7
**Preventive actions undertaken by mothers simultaneously**					
None	49.6	**60.9**	**45.0**	**46.6**	**50.1**
1 out of 3	25.1	**15.8**	**28.7**	**29.6**	**24.2**
2 out of 3	11.9	**10.7**	**12.4**	**11.7**	**12.0**
3 out of 3	13.4	**12.7**	**13.8**	**12.2**	**13.7**
At least one of these three actions	50.4	**39.1**	**55.0**	**53.4**	**49.9**

Notes: In bold, p<0.05 (differences between households’ locations);

^†^Rural high exposure, rural areas with large streams, lakes and/or stagnant water reservoirs

### Structural relationships (Table 4 and Fig 3)

**Table 4:** Generalized structural equation models: relationship between knowledge, attitudes, and practices and malaria transmission among under-five children.

Response variable*	Independent variable*	Unstandardized regression coefficient	Standardized regression coefficient	Odds ratio (OR)	95% Confidence interval	P value
Malaria^†^	Larval control actions	-0.49	-0.16	0.61	0.47–0.80	0.000
	ITN use	-0.55	-0.16	0.58	0.38–0.88	0.011
	Episode of illness	0.49	0.14	1.63	1.23–2.16	0.001
Larval control actions	Education	0.46	0.07	1.58	1.12–2.24	0.010
	Participation	0.61	0.07	1.84	1.18–2.86	0.007
	Knowledge	0.82	0.16	2.27	1.75–2.95	0.000
	Residence: (reference = rural high exposure)					
	Rural normal exposure	0.80	0.18	2.22	1.60–3.07	0.000
	Peri-urban	0.87	0.15	2.39	1.64–3.50	0.000
	Urban	0.41	0.29	1.50	1.08–2.07	0.014
ITN use^†^	Education	0.41	0.12	1.50	0.95–2.37	0.082
	Participation	0.91	0.20	2.48	1.29–4.76	0.006
	Episode of illness	0.30	0.13	1.35	1.02–1.79	0.038
Mothers’ knowledge about malaria	Education	0.55	0.06	1.73	1.07–2.78	0.025
Mothers’ participation in malaria information sessions	Residence (reference = rural high exposure)					
	Rural normal exposure	-0.19	-0.00	0.83	0.50–1.39	0.481
	Peri-urban	-1.10	-0.00	0.33	0.15–0.74	0.006
	Urban	-1.54	-0.00	0.21	0.11–0.41	0.000
Episode of illness	Residence (reference = rural high exposure)					
	Rural normal exposure	-0.55	-0.12	0.58	0.42–0.80	0.001
	Peri-urban	-0.49	-0.08	0.62	0.42–0.90	0.013
	Urban	-0.17	-0.12	0.84	0.62–1.15	0.275

Notes: ITN, Insecticide-treated net;

*Mothers’ variables: vector control actions, education (mothers who had gone to school), participation, and knowledge; Children’s variables: episode of illness, ITN use (child placed under an ITN the night before the survey); Households’ variable: residence;

^†^Two-level random intercept models (under-five children nested in households) and adjustment for age.

**Fig 3:**
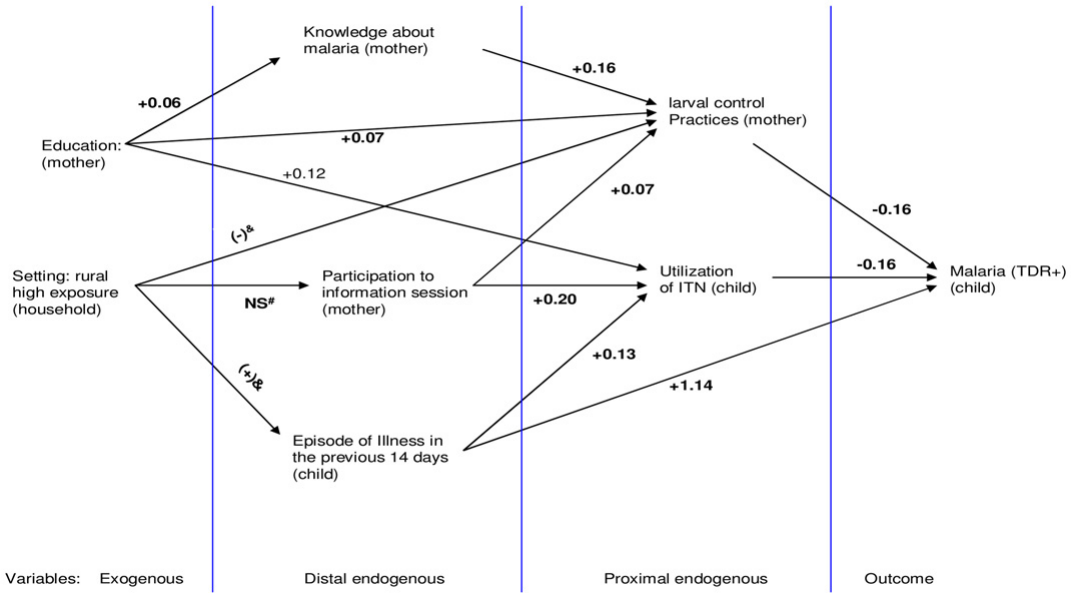
Generalized structural equation model for the relationship between prevention practices and malaria transmission. Legend: ^&^ Living in a highly exposed rural area increases the occurrence of illness and decreases vector control practices; ^#^Non-significant statistical association; in bold, standardized regression coefficients that are significantly different from zero; Education, mothers who had gone to school.

Mothers who had gone to school had better knowledge about malaria. They were also more likely to undertake domestic vector control practices and to place their children under bednets. Living in a highly exposed rural area significantly decreased the odds of undertaking domestic larval control actions. Domestic vector control actions were mostly undertaken by mothers with better knowledge about malaria. Mothers’ participation in information sessions on malaria directly increased both the adoption of domestic vector control actions and ITN use. Overall, the direct effects of mothers’ education and participation in information sessions on ITN use and on knowledge about vector control actions were high (standardized regression coefficients = +0.12, +0.20 and +0.16, respectively). Mothers’ education and participation in information sessions had comparable direct effects on domestic larval control practices (standardized regression coefficients = +0.07 for both relationships). The occurrence of illness among under-five children significantly increased ITN use (standardized regression coefficient = 0.13 and odds ratio = 1.35, p <0.05). Malaria prevalence was statistically lower among children in households where mothers had undertaken at least one vector control action or used ITNs. The effects of domestic vector control practices and ITN use on malaria prevalence were of the same magnitude: in both cases, there was a 0.16 standard deviation decrease in malaria prevalence for every standard deviation increase in vector control practices.

## Discussion

Half of the mothers had undertaken at least one environmental measure to control larval proliferation: cleaning of the house and its surroundings, eradication of larval sites, or elimination of hollow objects containing water or able to retain water. Similar results were reported in Ethiopia, where eradication of larval sites was reported by 47% of the respondents [19]. In Nigeria, on the other hand, cleaning of the surroundings and draining of stagnant water were reported by 8.5% and 17.5% of respondents, respectively [18]. Differences in the types of breeding sites, the intensity of information, and health education interventions may explain differences across studies [15]. Unlike in Nigeria, where there was a low level of formal education in rural communities [18], in Burkina Faso there are a variety of information, education, and communication activities to promote a healthy environment and control larval proliferation [2]. Nevertheless, these activities need to be reinforced, as 49.6% of the mothers surveyed had taken no action to reduce larval proliferation.

Studies seeking to identify the factors influencing mothers’ practice of at least one environmental measure to control larval proliferation have found that those who most often cleaned their house and its surroundings, eradicated larval sites, and eliminated hollow objects were primarily those who were educated and those who had, in the previous three months, attended information, education, and communication sessions targeting behaviour change, or those with a better knowledge of malaria. These results concur with those of previous studies [16, 27–28]. They show that promoting mothers’ education and encouraging their participation in awareness-raising sessions on malaria could contribute significantly to keeping the environment clean and healthy. The results also suggest that awareness campaigns to promote a cleaner and safer domestic environment targeting women in rural areas, where the risk of malaria transmission is high, are particularly effective. Factors related to households’ socioeconomic status, such as ownership of farm land or cattle and access to potable water, showed no impact on the adoption of environmental malaria prevention measures. On the other hand, the results of structural equation analyses showed that participation in malaria awareness-raising sessions and education both played a positive role in the use of bednets to protect children under five [27–28].

This study exploring the impact on malaria transmission of maintaining a clean and healthy home showed that domestic larval control practices tend to lower malaria prevalence among children under five. This impact was substantial, as the estimated reduction with adjusted odds ratios was 39%. This is a significant result in the Burkinabè context, where prevention tends to focus mainly on ITN use. It is also a major result in terms of malaria control in sub-Saharan Africa, where there is an abundance of natural or artificial larval habitats in homesteads, such as domestic waste, used tires, and other hollow objects where water can collect [10].

Large-scale larval source management has already resulted in the control of *Anopheles gambiae* in Brazil and Egypt decades ago [3–4]. Low malaria prevalence in north-eastern Ethiopia may explain why Abate et al. found no association between draining of stagnant water by respondents and malaria prevalence in households [19]. In the present study, domestic vector control practices and ITN use had equivalent effects on malaria prevalence. They had identical standardized regression coefficients. These results underscore the importance of implementing or reinforcing the promotion of domestic larval control actions and their integration into existing malaria prevention strategies (IRS and universal access to ITNs) [15].

Promoting community and household involvement in eradicating accessible larval breeding sites in homesteads has substantial advantages. First, contrary to ITNs and IRS, eradication of domestic larval sources is effective against both indoor and outdoor biting [34]. Second, resistance to insecticides and changes in adult mosquitoes’ behaviour are lessening the protective effects of ITNs and IRS. Multiple insecticide resistances have been reported in Burkina Faso and elsewhere in sub-Saharan Africa [35–37]. At the same time, adult mosquitoes such as *Anopheles gambiae* and *Anopheles funestus* (two of the most prevalent vectors in Burkina Faso) [38] tend to feed earlier, when people are still outdoors [37, 39–40]. Third, the domestic larval control practices investigated in this study, i.e., cleaning of the house and its surroundings, eradication of larval sites, and elimination of hollow objects that can contain and collect water, do not require additional financial resources. They can easily be undertaken by local communities only and implemented in conjunction with ITN use and IRS [7]. Finally, encouraging women to clean their homes, cover water containers, eliminate domestic waste, etc., can only improve hygiene and reduce households’ overall health risks. Currently, national malaria control programs place a great deal of emphasis on universal bednet access and tend to neglect raising awareness among health workers and families regarding maintaining a clean and healthy environment [41–42].

### Potential limitations

Malaria prevention practices were investigated in face-to-face interviews, which could have introduced a social desirability bias. Some mothers may have overestimated the extent to which they cleaned the house and its surrounding, eliminated hollow objects and stagnant water, and placed their under-five children under bednets. However, certain factors may have moderated the impact of this potential social desirability bias on the results [43–44]. The surveyors for the study were properly trained in qualitative research. They visited the mothers regularly, and the mothers knew that their responses did not, in any way, affect the services they received. Moreover, the mothers were assured that there were no right or wrong answers and were asked to answer the questions as honestly as possible. The causal hypotheses and the structural relationships diagram analyzed in this study may not be exhaustive. *A priori*, only the most essential factors were retained. It may be that other authors working in other circumstances would arrive at a somewhat different structural model. However, it is reassuring that: 1) nearly all the initially identified causal hypotheses were able to be reproduced; 2) the final model was highly consistent; and 3) the estimates regarding the scale and signs of the impacts were quite consistent.

## Conclusion

The results of this study showed that domestic practices involving cleaning the house and its surroundings, eradicating breeding sites, and eliminating hollow objects in which water might collect all play a significant and substantial role in preventing malaria transmission among children under five. In terms of scale, the impact on malaria prevention of maintaining a clean and healthy environment is comparable to that of using bednets. Yet the resources invested and attention paid to promoting bednet use are disproportionately much greater than those devoted to promoting domestic practices to foster a healthy environment. Thus, there is a real need for national malaria control and prevention programs to include and/or reinforce training activities for community health workers with the aim of promoting domestic practices to control larval proliferation.
